# Uncertainty-Aware and Lesion-Specific Image Synthesis in Multiple Sclerosis Magnetic Resonance Imaging: A Multicentric Validation Study

**DOI:** 10.3389/fnins.2022.889808

**Published:** 2022-04-26

**Authors:** Tom Finck, Hongwei Li, Sarah Schlaeger, Lioba Grundl, Nico Sollmann, Benjamin Bender, Eva Bürkle, Claus Zimmer, Jan Kirschke, Björn Menze, Mark Mühlau, Benedikt Wiestler

**Affiliations:** ^1^Department of Diagnostic and Interventional Neuroradiology, School of Medicine, Klinikum rechts der Isar, Technical University of Munich, Munich, Germany; ^2^Image-Based Biomedical Modeling, Technical University of Munich, Munich, Germany; ^3^Department of Diagnostic and Interventional Radiology, University Hospital Ulm, Ulm, Germany; ^4^Department of Diagnostic and Interventional Neuroradiology, Universitätsklinikum Tübingen, Tübingen, Germany; ^5^TUM-Neuroimaging Center, Klinikum rechts der Isar, Technical University of Munich, Munich, Germany; ^6^Department of Neurology, School of Medicine, Klinikum rechts der Isar, Technical University of Munich, Munich, Germany

**Keywords:** magnetic resonance imaging, neuroradiology, multiple sclerosis, deep learning – artificial neural network (DL-ANN), double inversion recovery (DIR), synthetic MRI, artificial intelligence (AI)

## Abstract

Generative adversarial networks (GANs) can synthesize high-contrast MRI from lower-contrast input. Targeted translation of parenchymal lesions in multiple sclerosis (MS), as well as visualization of model confidence further augment their utility, provided that the GAN generalizes reliably across different scanners. We here investigate the generalizability of a refined GAN for synthesizing high-contrast double inversion recovery (DIR) images and propose the use of uncertainty maps to further enhance its clinical utility and trustworthiness. A GAN was trained to synthesize DIR from input fluid-attenuated inversion recovery (FLAIR) and T1w of 50 MS patients (training data). In another 50 patients (test data), two blinded readers (R1 and R2) independently quantified lesions in synthetic DIR (synthDIR), acquired DIR (trueDIR) and FLAIR. Of the 50 test patients, 20 were acquired on the same scanner as training data (internal data), while 30 were scanned at different scanners with heterogeneous field strengths and protocols (external data). Lesion-to-Background ratios (LBR) for MS-lesions vs. normal appearing white matter, as well as image quality parameters were calculated. Uncertainty maps were generated to visualize model confidence. Significantly more MS-specific lesions were found in synthDIR compared to FLAIR (R1: 26.7 ± 2.6 vs. 22.5 ± 2.2 *p* < 0.0001; R2: 22.8 ± 2.2 vs. 19.9 ± 2.0, *p* = 0.0005). While trueDIR remained superior to synthDIR in R1 [28.6 ± 2.9 vs. 26.7 ± 2.6 (*p* = 0.0021)], both sequences showed comparable lesion conspicuity in R2 [23.3 ± 2.4 vs. 22.8 ± 2.2 (*p* = 0.98)]. Importantly, improvements in lesion counts were similar in internal and external data. Measurements of LBR confirmed that lesion-focused GAN training significantly improved lesion conspicuity. The use of uncertainty maps furthermore helped discriminate between MS lesions and artifacts. In conclusion, this multicentric study confirms the external validity of a lesion-focused Deep-Learning tool aimed at MS imaging. When implemented, uncertainty maps are promising to increase the trustworthiness of synthetic MRI.

## Introduction

Magnetic resonance imaging (MRI) plays a central role in the management of patients with multiple sclerosis (MS), a neuroinflammatory disease with rising incidence that remains the most common cause of non-traumatic disability in the young (GBD 2016 Multiple Sclerosis Collaborators, 2019). MRI techniques have been developed to detect specific aspects of MS pathophysiology; double inversion recovery (DIR) imaging is exemplary of a sequence that improves lesion detection, in particular within the juxtacortical region. Through numerous studies, the superiority of DIR compared to established MRI sequences such as T2w or fluid-attenuated inversion recovery (FLAIR) sequences in depicting inflammatory white matter lesions has been validated (Geurts et al., 2005; Wattjes et al., 2007). Lengthy acquisition times and high technical requirements have, however, hindered the widespread use of DIR.

Recently, it has been shown that synthesizing DIR images with generative adversarial networks (GANs), a deep learning (DL) architecture with great potential for image synthesis, is feasible and improves lesion detection compared to FLAIR and T2w sequences (Finck et al., 2020; Bouman et al., 2021). Nonetheless, and in particular as MS lesions typically are small, GANs are at risk to synthesize images of high morphologic similarity to the target image, while failing to translate the clinically important MS lesions. Domain knowledge, i.e., the ability of a GAN to learn about the pathology-specific anomalies it should map, might open the door for further customization and improvements in this regard. Various classification tasks, from the categorization of breast lesions to the detection of malignant thyroid nodules have thus already been improved by complementing a network’s training stage with domain knowledge (Feng et al., 2020; Avola et al., 2021). The underlying study is to our knowledge the first to investigate this knowledge-driven GAN approach in MS imaging.

The value of machine learning (ML) tools generally hinges on their ability to remain accurate when deployed to data that is of different structure from the training data, making multicentric validation a mandatory prerequisite. Also, building trust in artificial intelligence (AI) is oftentimes hindered because the decision-making process is concealed to the user who can only accept or discard a binary output (Asan et al., 2020). Hence, providing visibility into how an ML system makes predictions has become a major concern, especially in the medical domain (Quinn et al., 2022). This can be achieved either by providing insights into the “black-box” problem of DL systems that are inherently uninterpretable by the human operator or by designing networks that are inherently interpretable but generally less potent (i.e., linear regression, decision-trees). Neural networks are a hallmark of the “black-box” problem as decisions are made through nonlinear associations between input and output, thus remaining opaque to the human reader. Improved interpretability can be achieved by decreasing the complexity of such networks (i.e., reducing the amount of neural connections), at the potential cost of performance loss, or through uncertainty measurements of the decision-making process (Le et al., 2020). By providing uncertainty maps that quantify the decision-making confidence of a GAN, the acceptance of synthetic MRI by the medical community might be improved while also offering clearer insights into potential causes for a system’s malfunctioning. Uncertainty maps can be estimated by analysis of the variance across iterations during image synthesis, which has of late become an area of increasing interest (Gal and Ghahramani, 2015; Watson et al., 2019). Visualization of model confidence in GAN-mediated synthesis of MRI has been done before in tasks such as artificial motion-artifact inclusion or age prediction in fetal MRI (Shaw et al., 2020; Shi et al., 2020). In contrast to these works, we aim to quantify model confidence in translating areas of pathology that only constitute a small fraction of the generated data volume.

This study presents a refined GAN framework with an architecture that includes a task-specific training objective for MS lesion translation. We hypothesize that this GAN-based approach can provide synthetic, high-contrast DIR images from routinely acquired input FLAIR and T1w data, thereby removing the need for time-intensive acquisition of DIR. A special focus of this study is to evaluate this task-specific network for external validity in a multicenter dataset with scanners from different vendors and different acquisition details. To further provide an insight into the decision-making process of the GAN and guide the reviewing clinician toward potential artifacts, we calculated uncertainty maps that reflect the variance in image-to-image translation.

## Materials and Methods

### Patients

The study design was approved by the local IRBs and informed consent was obtained from all patients at their respective centers prior to scan acquisition.

### Training Data

Data for model training were retrospectively retrieved from 50 patients with diagnosed MS and included T1w (2:28 min), FLAIR (3:55 min), and DIR (6:31 min). All scans originated from the same scanner (Ingenia 3.0T, Philips Healthcare, Best, Netherlands). Sequence parameters were identical in all patients for T1w (TR of 9.0 ms, TE of 4.0 ms, flip angle of 8°, acquired in the sagittal plane with an isotropic voxel size of 1 mm^3^), FLAIR (TR of 4,800 ms, TE of 331 ms, TI of 1,650 ms, flip angle of 90°, acquired in the sagittal plane with an isotropic voxel size of 1 mm^3^), and DIR (TR of 5,500 ms, TE of 355.9 ms, TI of 2,550 ms and 2,990 ms, flip angle of 90°, acquired in the sagittal plane with an isotropic voxel size of 1.1 mm^3^).

### Testing Data

Sixty MRI scans from 50 consecutive patients (20:20:10 for centers 1:2:3, respectively) with diagnosed MS were included. For centers 1 and 2, 1 scan/patient was sampled, while baseline and follow-up exams for 10 patients from center 3 were considered. MRI data included T1w, FLAIR, and DIR and were acquired on both, 3.0T and 1.5T scanners. In detail, testing data from center 1 was acquired on the same hardware and using the same protocol as the training data (Ingenia 3.0T, Philips Healthcare, Best, Netherlands), testing data from center 2 originated from a different 3.0T scanner from the same manufacturer (Achieva 3.0T, Philips Healthcare, Best, Netherlands), and testing data from center 3 was acquired on 1.5T and 3.0T scanners from a different manufacturer (Skyra 3.0T, Avanto_fit 1.5T, and Aera 1.5T, Siemens Healthineers, Erlangen, Germany).

Sequence parameters for T1w, FLAIR, and DIR sequences were chosen according to the site-specific parameters optimized for routine clinical imaging and not modified during the retrieval period (Supplementary Table 1). Dichotomization of data from centers 1–3 was made to acknowledge the fact that data structure from (1) corresponded to the training data (prospectively referred to as “internal data”), while the data structure from (2) and (3) was unknown to the network (prospectively referred to as “external data”). Table 1 illustrates how the data was categorized for evaluation.

**TABLE 1:** Data from center 1 was acquired on the same hardware as training data and thus considered to be of known structure (= internal data).

Data class (number of image sets)	Classes for study evaluation
Training data (*n* = 50)	
Test data from (1) (*n* = 20)	Internal data (Known data structure)
Test data from (2) (*n* = 20)	External data (Unknown data structure)
Test data from (3) (*n* = 20)	External data (Unknown data structure)

*In analogy, data from centers 2 and 3 were acquired on different hardware and considered to be of unknown structure (= external data).*

### Double Inversion Recovery Image Synthesis

#### Network Architecture

**FIGURE 1:**
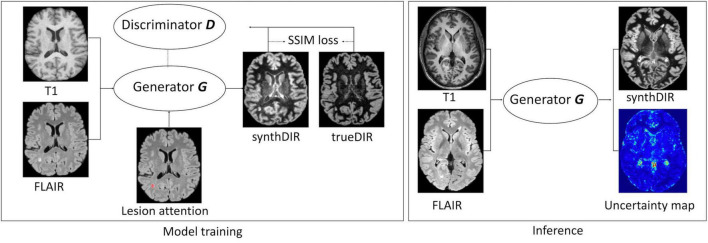
Architecture, training process, and inference of the image synthesis task. The image Generator G uses the combination of FLAIR and T1w as input to generate synthDIR. The additional supervision from the lesion maps in the training stage drives an enhanced translation of MS-specific lesions (lesion attention). The feedback on the similarity between synthDIR and trueDIR is given by the Discriminator D and a structure similarity loss function and it updates the network weights until the loss function to discern both image pairs is minimal. During the inference stage, the trained generator G can generate the synthDIR and an uncertainty map showing the confidence of the output relating to each voxel. Uncertainty maps are calculated from the voxel-wise variances in signal intensities, as explained in the section “Materials and Methods”.

Our GAN extends the existing “pix2pix” method (Isola et al., 2017) and is trained to synthesize a target image *y* (resembling the true target image *Y*) given a set of input images *X* and a lesion segmentation mask *S*. In this setting, two networks compete with each other: The generator *G* is based on a U-Net architecture and synthesizes the target DIR images (synthDIR) from two input images (T1w and FLAIR), while the discriminator *D* tries to determine if a given DIR image is synthetic (synthDIR) or physically acquired (trueDIR). The network architecture and training process of the GAN are given in Figure 1. Importantly, the input of T1 and FLAIR images are fed to U-Net to generate DIR images while the lesion mask is only used to compute additional lesion-specific loss during the training stage (see below). Thus the lesion segmentation mask *S* is not required during inference.

#### Loss Functions

The discriminator gives the judgment about how realistic the local structures are (called “Patch GAN”), and is patch-based and driven by a least-square error (L2) loss function (Mao et al., 2019). The generator is trained on a composite loss function based on (a) the reconstruction error between the synthesized image and the target image using SSIM and (b) the output of the discriminator when judging if a given image is either ground truth or synthetic. In addition to an SSIM, a peculiarity of our model is that an additional loss focusing on the successful translation of MS lesions was developed. In order to focus the model on MS lesions (which only make up a minority of voxels in an image), an additional L1 loss term is calculated between the true and synthetic DIR images after multiplying both images with the lesion segmentation mask *S*, thus only considering the translation of MS lesions for this part of the loss. The image reconstruction loss for the generator *G*, the loss function for the discriminator *D*, and the total loss function were formulated as follows, respectively:


(1) $${ℒ}_{r\,e\,c\,o\,n\,s}=1-S\,S\,I\,M\,\left(Y,G\,\left(X\right)\right)+\lambda_{1}*||\left(Y-G\,\left(X\right)\right)⊙S_{1}||$$

(2) $${ℒ}_{D}={𝔼}_{X}\,\left\{{||1-D\,\left(X\right)||}_{2}\right\}$$

(3) $${ℒ}_{t\,o\,t\,a\,l}=\lambda_{2}*{ℒ}_{r\,e\,c\,o\,n\,s}+{ℒ}_{D}$$

Here, λ_*1*_ and λ_*2*_ are hyper-parameters and set to 1 and 10, respectively, which balances the two loss components.

#### Optimization

**FIGURE 2:**
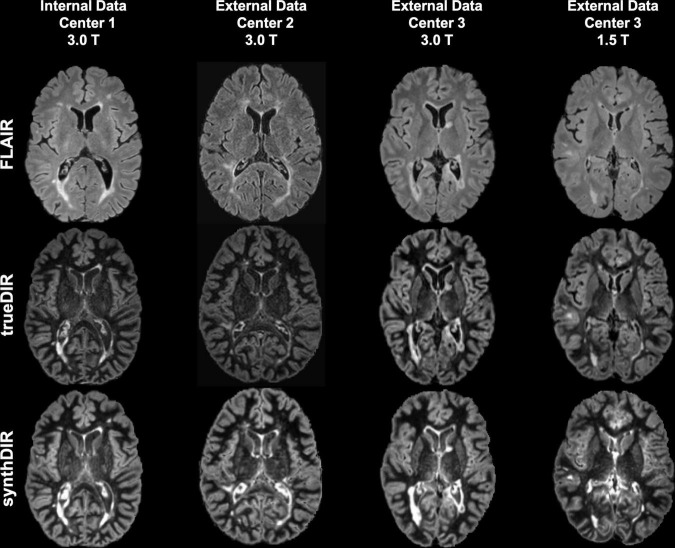
Exemplary images of FLAIR, trueDIR, and synthDIR for all centers and scanners.

The input and output images were co-registered, skull-stripped, linearly transformed into the MNI152 space, and resampled to 1 mm isotropic resolution. As excellent correlation between automated and manual segmentation performance has been shown before, lesion segmentation maps were created using the Lesion Segmentation Tool (LST) (Schmidt et al., 2012). By including domain knowledge (in the form of lesion segmentation on FLAIR images) into the image translation during training, we enforced the model to pay attention to the lesion area by minimizing the difference between ground-truth images and synthetic images. In practice, such segmentation maps can be also provided by manual segmentation or other automated lesion segmentation tools (Schmidt et al., 2012; Li et al., 2018). Exemplary cases of all investigated sequences are shown in Figure 2. Training was carried out with a batch size of 1 for a total of 150 epochs, using the Adam optimizer with a learning rate of 0.001. During training, random intensity (gamma correction and gaussian blurring) and spatial (shifting and flipping) augmentations were performed. The best-performing model was selected using an internal validation set consisting of 10% of the training images.

The generated model is publicly available at https://figshare.com/articles/software/synthDIR/16607831.

### Expert Readings

A dataset of 180 scans, comprising 60 sets each for FLAIR, synthDIR, and trueDIR, was investigated for lesion counts by two neuroradiologists (R1 with 5 years of experience in neuroradiological imaging, R2 with 3 years of experience in neuroradiological imaging) in a random order. Readers were blinded to scanner types and sequence labels. The number of juxtacortical (JC), periventricular (PV), infratentorial (IT), and subcortical (SC) lesions, in accordance with the 2017 McDonald criteria, were counted (Thompson et al., 2018). JC, PV, and IT lesions were considered to be MS-specific (Thompson et al., 2018). Albeit known to constitute different pathophysiological entities, we did not differentiate between cortical and juxtacortical lesions as this approach best reflects current guidelines (Bo et al., 2003; Thompson et al., 2018).

### Quantitative Lesion Analysis and Uncertainty Maps

To quantitatively assess lesion translation, we calculated lesion-to-background ratios (LBR). Therefore, lesions on FLAIR and T1w images were segmented using LST, and tissue segmentation of T1w images was performed using ANTs Atropos (Avants et al., 2011). For comparison of LBR, GAN iterations with and without the above-stated lesion-specific loss function were computed.

From the segmentation maps, the lesion-to-background ratio was calculated as:


(4) $$L\,B\,R=\frac{M\,e\,a\,n\,S\,i\,g\,n\,a\,l_{l\,e\,s\,i\,o\,n}}{M\,e\,a\,n\,S\,i\,g\,n\,a\,l_{N\,A\,W\,M}}$$

Here, NAWM refers to “normal appearing white matter,” i.e., non-lesioned white matter. From lesion segmentation maps and corresponding annotations in the NAWM, the mean signal intensity was extracted from DIR, FLAIR, and synthDIR images.

To estimate the GAN’s uncertainty in generating synthDIR images, we performed variational inference during the test time by using dropout sampling. We added a dropout layer (dropout rate of 0.3) to the second-last layer of the U-Net and calculated 100 synthDIR images per input (Gal and Ghahramani, 2015). From these 100 iterations, we calculated the variance of voxel-wise intensities, resulting in the uncertainty map for visual inspection.

### Statistical Analysis

Lesion counts were compared with a Wilcoxon signed-rank test to account for non-Gaussian distribution and paired data. LBR was compared with a paired *t*-test. Similarity of synthDIR and trueDIR was furthermore quantitatively assessed by the SSIM (Wang et al., 2004). For pixelwise comparisons, peak signal-to-noise ratio (PSNR) was calculated. Interrater agreement was assessed with the intraclass correlation coefficient (ICC; use of single measurements for absolute agreement in a two-way random model) and the related 95% confidence interval (95% CI). Statistical computations were performed with SPSS software (SPSS Statistics for Windows, version 25.0; IBM, Armonk, NY, United States). A *p*-value < 0.05 was considered statistically significant.

## Results

### Interrater Agreement

Consistency between both readers was excellent with ICCs for all specific (JC + PV + IT) lesions ranging from 0.91 (95% CI: 0.85; 0.94) in FLAIR to 0.90 (95% CI: 0.84; 0.94) in synthDIR and 0.89 (95% CI: 0.83; 0.94) in trueDIR.

### Lesion Counts

The study endpoint to improve depiction of MS specific lesions in synthDIR compared to FLAIR was met by both readers [26.7 ± 2.6 vs. 22.5 ± 2.2 (*p* < 0.0001) in R1 and 22.8 ± 2.2 vs. 19.9 ± 2.0 (*p* = 0.0005) in R2]. TrueDIR outperformed FLAIR in counts of MS-specific lesions [28.6 ± 2.9 vs. 22.5 ± 2.2 (*p* < 0.0001) in R1 and 23.3 ± 2.4 vs. 19.9 ± 2.0 (*p* < 0.0001) in R2]. While trueDIR remained superior to synthDIR in the depiction of MS-specific lesions in R1 [28.6 ± 2.9 vs. 26.7 ± 2.6 (*p* = 0.0021)], both image sets were of comparable diagnostic value in R2 [23.3 ± 2.4 vs. 22.8 ± 2.2 (*p* = 0.98)]. Table 2 provides details on total and region-specific lesion counts for the study cohort.

**TABLE 2:** Lesion counts for all locations and both readers.

	All specific	*P*	PV lesions	*P*	JC lesions	*P*	IT lesions	*P*	SC lesions	*P*
**Reader 1**										
FLAIR vs. synthDIR	22.5 ± 2.2 vs. 26.7 ± 2.6	<0.0001	12.0 ± 1.2 vs. 13.9 ± 1.4	<0.0001	8.7 ± 1.2 vs. 10.8 ± 1.5	<0.0001	1.9 ± 0.4 vs. 2.2 ± 0.4	0.043	10.6 ± 1.3 vs. 10.4 ± 1.2	0.82
FLAIR vs. trueDIR	22.5 ± 2.2 vs. 28.6 ± 2.9	<0.0001	12.0 ± 1.2 vs. 13.9 ± 1.4	<0.0001	8.7 ± 1.2 vs. 12.3 ± 1.7	<0.0001	1.9 ± 0.4 vs. 2.4 ± 0.4	0.0002	10.6 ± 1.3 vs. 10.9 ± 1.4	0.36
SynthDIR vs. trueDIR	26.7 ± 2.6 vs. 28.6 ± 2.9	0.0021	13.9 ± 1.4 vs. 13.9 ± 1.4	0.91	10.8 ± 1.5 vs. 12.3 ± 1.7	<0.0001	2.2 ± 0.4 vs. 2.4 ± 0.4	0.33	10.4 ± 1.2 vs. 10.9 ± 1.4	0.66
**Reader 2**										
FLAIR vs. synthDIR	19.9 ± 2.0 vs. 22.8 ± 2.2	0.0005	10.5 ± 1.0 vs. 12.4 ± 1.1	0.0004	7.8 ± 1.2 vs. 8.5 ± 1.3	0.18	1.5 ± 0.3 vs. 1.9 ± 0.3	0.024	13.5 ± 1.9 vs. 10.5 ± 1.5	<0.0001
FLAIR vs. trueDIR	19.9 ± 2.0 vs. 23.3 ± 2.4	<0.0001	10.5 ± 1.0 vs. 12.2 ± 1.2	0.0014	7.8 ± 1.2 vs. 9.7 ± 1.5	0.0028	1.5 ± 0.3 vs. 1.5 ± 0.3	0.99	13.5 ± 1.9 vs. 10.5 ± 1.6	<0.0001
SynthDIR vs. trueDIR	22.8 ± 2.2 vs. 23.3 ± 2.4	0.98	12.4 ± 1.1 vs. 12.2 ± 1.2	0.26	8.5 ± 1.3 vs. 9.7 ± 1.5	0.068	1.9 ± 0.3 vs. 1.5 ± 0.3	0.03	10.5 ± 1.5 vs. 10.5 ± 1.6	0.70

*PV, periventricular; JC, juxtacortical; IT, infratentorial; SC, subcortical; FLAIR, fluid-attenuated inversion recovery; trueDIR, real double inversion recovery; synthDIR, synthetic double inversion recovery.*

Analysis of lesion counts as a function of scanner types revealed comparable effects independent of the structure of input data (internal or external). Hence, significant improvements in lesion counts were noted in synthDIR vs. FLAIR for both readers in external data [27.1 ± 3.4 vs. 22.6 ± 2.8 (*p* < 0.0001) in R1; 25.1 ± 2.9 vs. 21.5 ± 2.6 (*p* = 0.0007) in R2] and for R1 in internal data [26.6 ± 4.3 vs. 22.2 ± 3.6 (*p* = 0.0029) in R1; 18.1 ± 2.6 vs. 16.6 ± 2.6 (*p* = 0.27) in R2]. In external data, a slight improvement in lesion conspicuity was noted in trueDIR vs. synthDIR for R1 [28.9 ± 3.7 vs. 27.1 ± 3.4 (*p* = 0.011)] but not for R2 [25.6 ± 3.3 vs. 25.1 ± 2.9 (*p* = 0.90)]. Table 3 provides lesion counts as a function of data source.

**TABLE 3:** Counts of MS-specific lesions for FLAIR, trueDIR, and synthDIR as a function of data source.

	All	*P*	Internal data	*P*	External data	*P*
**Reader 1**						
FLAIR vs. synthDIR	22.5 ± 2.2 vs. 26.7 ± 2.6	<0.0001	22.2 ± 3.6 vs. 26.6 ± 4.3	0.0029	22.6 ± 2.8 vs. 27.1 ± 3.4	<0.0001
FLAIR vs. trueDIR	22.5 ± 2.2 vs. 28.6 ± 2.9	<0.0001	22.2 ± 3.6 vs. 27.9 ± 4.6	0.0001	22.6 ± 2.8 vs. 28.9 ± 3.7	<0.0001
SynthDIR vs. trueDIR	26.7 ± 2.6 vs. 28.6 ± 2.9	0.0021	26.6 ± 4.3 vs. 27.9 ± 4.6	0.086	27.1 ± 3.4 vs. 28.9 ± 3.7	0.011
**Reader 2**						
FLAIR vs. synthDIR	19.9 ± 2.0 vs. 22.8 ± 2.2	0.0005	16.6 ± 2.6 vs. 18.1 ± 2.6	0.27	21.5 ± 2.6 vs. 25.1 ± 2.9	0.0007
FLAIR vs. trueDIR	19.9 ± 2.0 vs. 23.3 ± 2.4	<0.0001	16.6 ± 2.6 vs. 18.6 ± 2.7	0.027	21.5 ± 2.6 vs. 25.6 ± 3.3	0.0001
SynthDIR vs. trueDIR	22.8 ± 2.2 vs. 23.3 ± 2.4	0.98	18.1 ± 2.6 vs. 18.6 ± 2.7	0.87	25.1 ± 2.9 vs. 25.6 ± 3.3	0.90

*FLAIR, fluid-attenuated inversion recovery; trueDIR, real double inversion recovery; synthDIR, synthetic double inversion recovery.*

**FIGURE 3:**
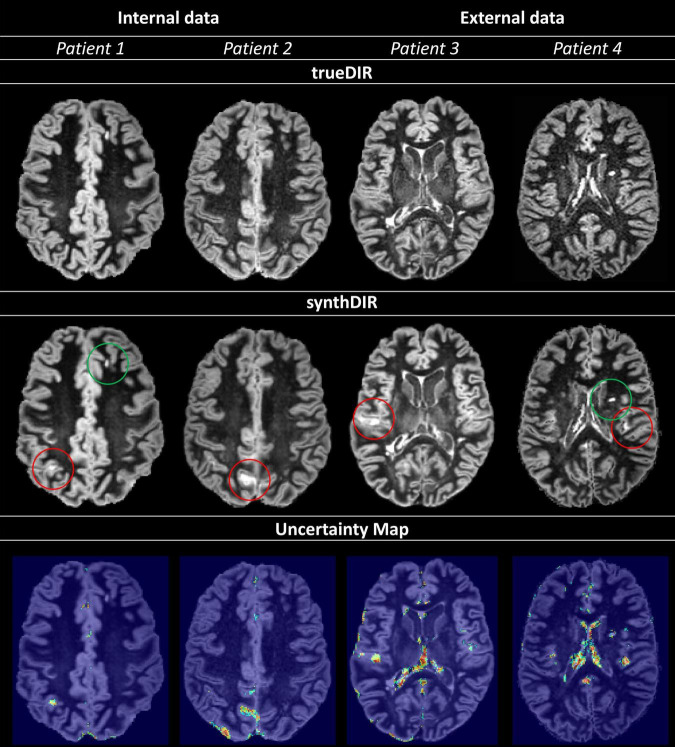
Uncertainty maps provide relevant information regarding the validity of voxel-to-voxel translation; increases in uncertainty are scaled from blue to green. Circled in red (Patients 1–4) are hyperintensities in synthDIR without correlation in trueDIR and easily recognized as areas of high variance in the corresponding uncertainty maps, allowing for their identification as artifacts from the synthesis task. On the other hand, true-positive lesions are readily identified as regions with either no (patient 1 – green circle in synthDIR) or low (patient 4 – green circle in synthDIR) values of uncertainty. Hence, interpretation of synthDIR and decision-making on the veracity of lesions is facilitated through uncertainty maps.

To increase the clinical reliability of synthDIR images, voxel-wise uncertainty maps from 100 forward runs using test-time dropout for Bayesian approximation were evaluated. For the majority of lesions, a high model confidence was observed, i.e., lesions were not highlighted in the uncertainty maps. On the other hand, artificial hyperintensities in synthetic images were readily identified by the high model uncertainty on these maps. Figure 3 provides examples on how uncertainty maps allow to discern true-positive lesions from false-positive hyperintensities in synthDIR.

### Quantitative Image Analysis

Similarity between trueDIR and synthDIR was highest in internal data, as shown by an SSIM of 0.967 ± 0.012, closely followed by external data (3) and (2) with still excellent SSIM-values of 0.950 ± 0.012 and 0.941 ± 0.010, respectively. For synthDIR, PSNR was highest in internal data at 29.2 ± 1.6 dB and decreased to 25.6 ± 1.1 dB in external data (3). Table 4 provides detailed values for quantitative image metrics.

**TABLE 4:** Image-wise (SSIM) and voxel-wise (PSNR) comparative metrics for synthDIR and trueDIR.

	SSIM (trueDIR – synthDIR)	PSNR (dB) (trueDIR – synthDIR)	LBR FLAIR	LBR trueDIR	LBR synthDIR	LBR synthDIR w/o LFL
All	0.954 ± 0.016	27.2 ± 2.2	1.52 ± 0.49	2.86 ± 0.65	2.80 ± 0.67	2.69 ± 0.66
Internal data	0.967 ± 0.012	29.2 ± 1.64	1.45 ± 0.06	2.80 ± 0.33	2.86 ± 0.34	2.68 ± 0.30
External data (2)	0.941 ± 0.010	25.8 ± 1.12	1.65 ± 0.12	3.01 ± 0.41	3.35 ± 0.50	3.31 ± 0.45
External data (3)	0.950 ± 0.012	25.6 ± 1.08	1.46 ± 0.86	2.78 ± 1.00	2.19 ± 0.56	2.07 ± 0.50

*LBR are given for FLAIR, trueDIR, synthDIR, as well as for synthDIR generated by a GAN iteration without the lesion-focused loss function (synthDIR w/o LFL). Results are given for internal data, as well as external data (2) and (3). SSIM, structural similarity index measure; PSNR, peak signal-to-noise ratio; LBR, lesion-to-background ratio; LFL, lesion-focused loss; trueDIR, real double inversion recovery; synthDIR, synthetic double inversion recovery.*

### Effects of Lesion-Focused Loss Function

To assess the benefit of the lesion-specific loss function during image synthesis, LBR were compared between FLAIR, trueDIR, synthDIR, as well as synthDIR generated by a network iteration without the lesion-specific loss. Both versions of synthDIR, irrespective if additional loss was included or not, exceeded input FLAIR in LBR (data given in Table 4).

**FIGURE 4:**
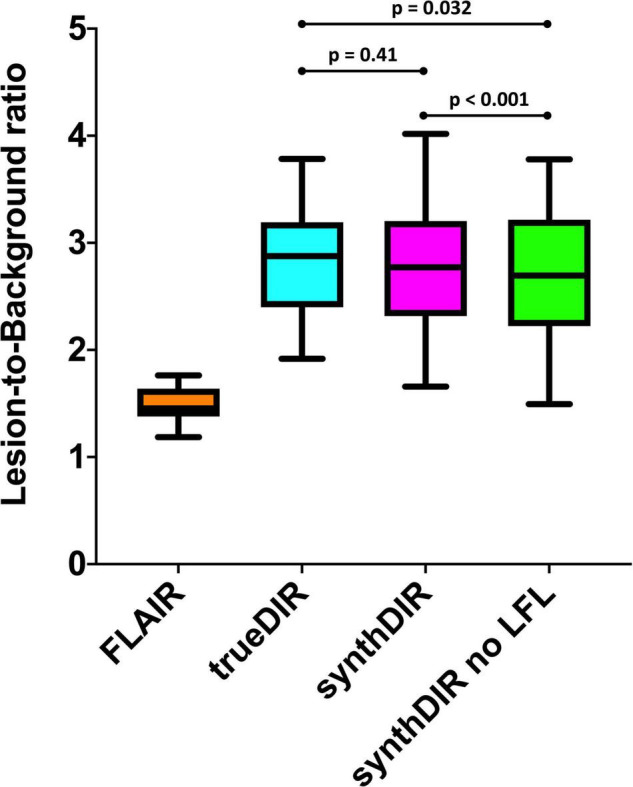
Lesion-to-background ratios for FLAIR, trueDIR, and synthDIR. Additionally, LBR was calculated for synthDIR generated by a GAN-iteration without the lesion-focused loss (synthDIR no LFL). Of note, LBR was significantly higher in synthDIR compared to synthDIR without LFL, confirming the hypothesis that domain knowledge can be improved through LFL. While there was no significant difference in LBR between synthDIR and trueDIR (*p* = 0.41), LBR of synthDIR without LFL remained inferior to the LBR of trueDIR (*p* = 0.032). LBR, lesion-to-background ratio; LFL, lesion-focused loss.

Of note, LBR was significantly lower in synthDIR generated by the version without lesion-focused loss compared to the version of synthDIR benefiting from lesion-focused loss (2.69 ± 0.66 vs. 2.80 ± 0.67, *p* < 0.001). While synthDIR achieved a comparable LBR to trueDIR (2.80 ± 0.67 vs. 2.86 ± 0.65, *p* = 0.41), this effect faded if synthDIR was generated without lesion-focused loss (2.69 ± 0.66 vs. 2.86 ± 0.65, *p* = 0.032) (as shown in Figure 4).

## Discussion

Medical imaging has benefited greatly from DL advances that gave birth to a panoply of systems aimed at tasks ranging from disease detection to image synthesis and artifact reduction (Emami et al., 2018; Rajpurkar et al., 2018; Liang et al., 2019). We here validated a GAN that has been fine-tuned to the translation of MS-specific white matter lesions while aiming to remain generalizable to external data. We further explored the concept of uncertainty maps to illustrate how trustworthy the network is in image-to-image translation. Such maps can provide important support to decide on the veracity of findings in synthetic images and help the radiologist to detect artifacts resulting from the synthesis task.

Comparison of the network’s performance in internal and external data showed that significantly more MS-specific lesions could be found in synthDIR compared to the FLAIR sequence that was used as input, irrespective of the data origin. Approximately 20% more MS-specific lesions were thus depictable in synthDIR, a magnitude of difference that is of obvious clinical interest, especially in patients with low lesion counts. While other surrogates of MS activity have been explored, depiction of new inflammatory plaques is still considered the hallmark of disease monitoring in MS (Filippi et al., 2001; Chard et al., 2003; Wattjes et al., 2015). Also, lesion load has been shown to directly correlate with future disability and, if properly detected and reliably quantified, might therefore prompt escalation of disease-modifying therapy (Calabrese et al., 2010; Popescu et al., 2011).

Domain knowledge, i.e., the ability to learn about pathology-specific image findings, is promising to further augment the clinical utility of DL tools (Yuan et al., 2019). The improved lesion translation that our GAN achieved by including a lesion-focused loss function hints at the potential of domain knowledge to further customize synthetic imaging. To highlight this, we showed that LBR in synthDIR was non-inferior to LBR in trueDIR only if the GAN was complemented by a lesion-focused loss.

The ability of synthDIR to outperform FLAIR, a sequence still considered gold-standard in MS imaging, has been shown for a multi-modal input (T1w, T2w, and FLAIR) in a monocentric setting (Finck et al., 2020; Bouman et al., 2021). In doing so, relevant reductions in scan times are feasible as the physical acquisition of 3D and isotropic DIR may take up to 7 min (Eichinger et al., 2019). While other methods, such as sparse sampling, have previously achieved scan time reductions for DIR, a GAN-based approach might be advantageous as it works on existing data and thus does not need to be prospectively deployed (Eichinger et al., 2019). This offers the potential advantage to augment the diagnostic value of existing studies and, hence, to render longitudinal follow-up exams more conclusive.

Albeit accurate in their output, neural networks generally fail to provide insight into the decision-making process, the so-called “black-box problem.” Rendering this process more transparent is crucial for the acceptance of said networks and can, in theory, be achieved by providing methods to interpret the “black-box,” or by designing models that are inherently more transparent in their functioning (Rudin, 2019; Arun et al., 2020). In GANs specifically, one potential bias in trying to match the (lesion) distribution in the target domain (trueDIR) is that features (lesions) with no correlation in source data might be erroneously mapped, a phenomenon commonly referred to as “hallucination.” To verify lesion veracity we therefore introduced the concept of uncertainty maps that highlight the voxel-wise aleatoric variance taking place during image translation. Hence, the ability to compare hyperintensities in synthDIR to their respective uncertainty mappings can reduce the risk of false-positive findings, i.e., misinterpretation of constructed lesions in the synthetic image data. Figure 3 illustrates how MS lesions can thus be separated from artifacts according to their voxel-wise intensity variance. As erroneous mappings remain an intrinsic limitation of GANs, their future deployment might benefit greatly from the calculation of uncertainty maps that are displayed in parallel to synthetic images.

A limitation of this approach is that having to reference synthDIR, along with the uncertainty maps adds complexity to the longitudinal interpretation of clinical MRI. Furthermore, comparison of synthDIR and trueDIR via autosegmentation techniques might have provided more objective lesion counts in this study. However, as our GAN was designed to provide synthetic data for clinical use, we opted for manual lesion counts as this best reflects the clinical reality. Future iterations of synthDIR might furthermore mitigate the wide disparities in lesion counts that we noticed especially in SC lesions. Also, prospective investigations should explore the feasibility to generate a GAN targeted to create synthDIR while using even fewer, potentially only one input modality. At last, we tested for generalizability by including three centers with differing hardware. Future investigations would benefit from the inclusion of more centers and readers, as our results demonstrate equivalence of synthDIR to trueDIR for only one of the two neuroradiologists.

## Conclusion

Our findings confirm the use-case and external validity of a DL tool targeted at improving MRI in patients with MS. Our study demonstrates both, the utility of lesion-focused learning to improve domain adaption, as well as the potential benefit of uncertainty maps to help gain trust in GANs and make informed medical decisions. Presumably, wider deployment of these tools could prove beneficial in MS where treatment decisions are heavily relying on MRI findings.

## Data Availability Statement

The datasets presented in this article are not readily available because of National Data Protection Law. Requests to access the datasets should be directed to TF, tom.finck@tum.de.

## Ethics Statement

The studies involving human participants were reviewed and approved by the Ethikkommission Klinikum rechts der Isar. The patients/participants provided their written informed consent to participate in this study.

## Author Contributions

BW, HL, BM, CZ, JK, TF, LG, and MM conceived and designed the project. BB and EB conceived the study and contributed external datasets. HL, BM, and BW designed the GAN. NS and SS performed the experiments. TF and BW prepared the writing of the first draft and performed the statistical analyses. TF prepared the figures and tables. All authors reviewed the first draft of the manuscript, contributed to the article, and approved the final manuscript draft.

## Conflict of Interest

The authors declare that the research was conducted in the absence of any commercial or financial relationships that could be construed as a potential conflict of interest.

## Publisher’s Note

All claims expressed in this article are solely those of the authors and do not necessarily represent those of their affiliated organizations, or those of the publisher, the editors and the reviewers. Any product that may be evaluated in this article, or claim that may be made by its manufacturer, is not guaranteed or endorsed by the publisher.

## Supplementary Material

Supplementary Figure 1 Network architecture: **(A)** The U-Net generator used to produce a synthetic DIR image from FLAIR and T1 input images. **(B)** The patch-based discriminator which receives as input both the source image (T1 and FLAIR) and either a real or synthetic DIR. The discriminator is patch-based. ks, kernel size.

## Abbreviations

MRI: magnetic resonance imaging
MS: multiple sclerosis
DIR: double inversion recovery
FLAIR: fluid-attenuated inversion recovery
GAN: generative adversarial network
DL: deep learning
ML: machine learning
AI: artificial intelligence
synthDIR: synthetic double inversion recovery
trueDIR: physically acquired double inversion recovery
SSIM: structural similarity index measure
LST: lesion segmentation tool
JC: juxtacortical
PV: periventricular
IT: infratentorial
SC: subcortical
LBR: lesion-to-background ratios
LFL: lesion-focused loss
NAWM: normal appearing white matter
PSNR: peak signal-to-noise ratio
ICC: intraclass correlation coefficient.
