# Supplementary figures and images for: Uncertainty-Aware and Lesion-Specific Image Synthesis in Multiple Sclerosis Magnetic Resonance Imaging: A Multicentric Validation Study
Source: Front Neurosci. 2022 Apr 26;16:889808. doi: 10.3389/fnins.2022.889808 (PMC9087732; doi:10.3389/fnins.2022.889808)

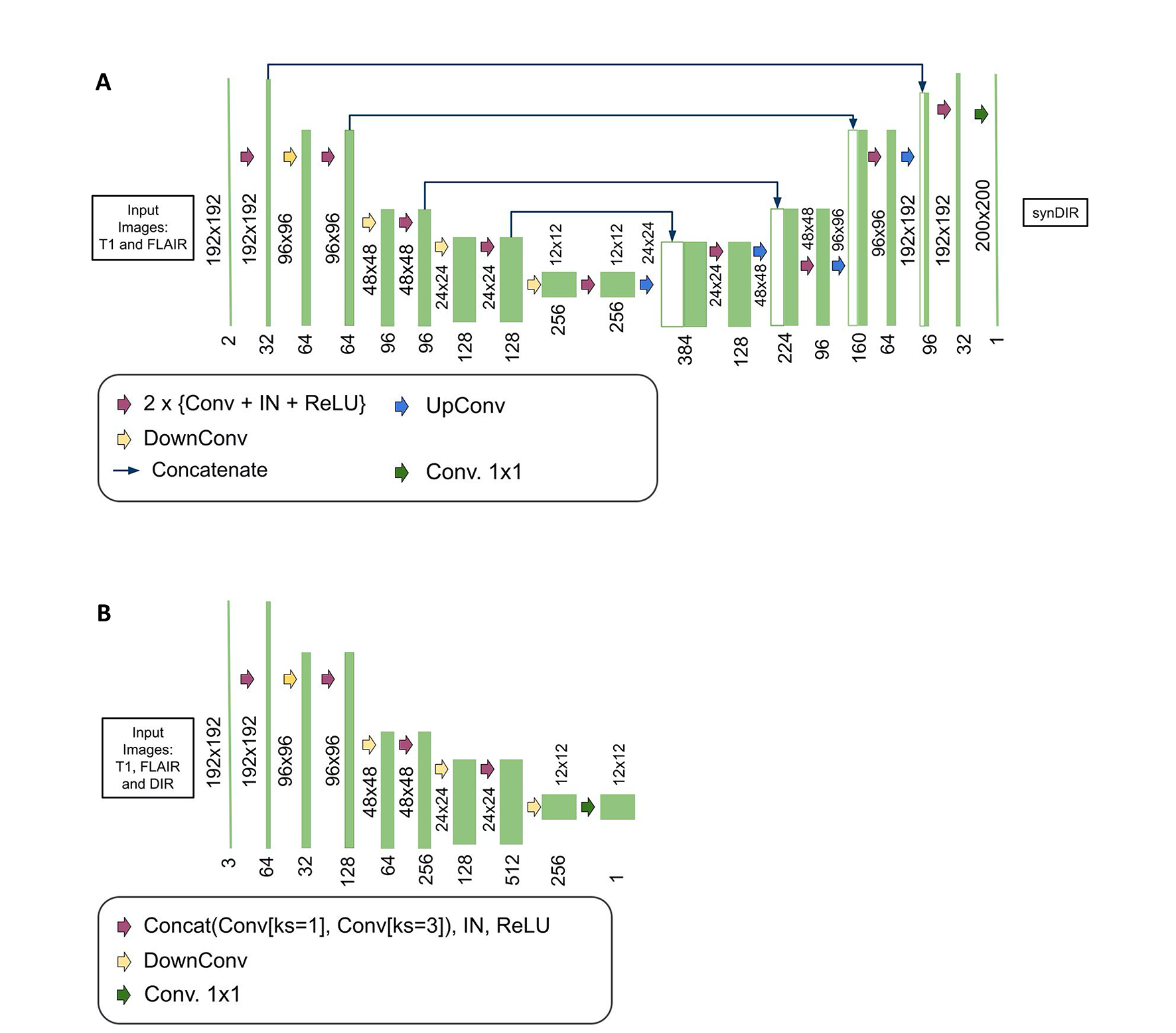

Supplement: Supplementary Figure 1 — Network architecture: (A) The U-Net generator used to produce a synthetic DIR image from FLAIR and T1 input images. (B) The patch-based discriminator which receives as input both the source image (T1 and FLAIR) and either a real or synthetic DIR. The discriminator is patch-based. ks, kernel size. [file Image_1.JPEG]
