# Supplementary material for: Uncertainty-Aware and Lesion-Specific Image Synthesis in Multiple Sclerosis Magnetic Resonance Imaging: A Multicentric Validation Study
Source: Front Neurosci. 2022 Apr 26;16:889808. doi: 10.3389/fnins.2022.889808 (PMC9087732; doi:10.3389/fnins.2022.889808)
Supplement: Supplementary file 2 [file Table_1.docx]

|  | Philips Achieva (3T) | | | Siemens Avanto_fit (1.5T) | | | Siemens Skyra (3T) | | | Siemens Aera (1.5T) | | |
| --- | --- | --- | --- | --- | --- | --- | --- | --- | --- | --- | --- | --- |
|  | T1 | FLAIR | DIR | T1 | FLAIR | DIR | T1 | FLAIR | DIR | T1 | FLAIR | DIR |
| Repetition time (ms) | 9 | 10000 | 5500 | 1280 | 5000 | 7500 | 2300 | 5000 | 7500 | 1280 | 5000 | 7500 |
| Echo time (ms) | 4 | 140 | 322 | 2.36 | 337 | 311 | 2.32 | 387 | 320 | 2.36 | 335 | 310 |
| Inversion time (ms) |  | 2750 | 2550/2990 | 660 | 1800 | 3000/3450 | 900 | 1800 | 3000/3450 | 660 | 1800 | 3000/3450 |
| Flip angle | 8° | 90° | 90° | 15° | 120° (vfl) | 120° (vfl) | 8° | 120° (vfl) | 120° (vfl) | 15° | 120° (vfl) | 120° (vfl) |
| Acquisition pane and voxel size | Sag., 1mm^3^ | Sag., 1mm^3^ | Sag., 1mm^3^ | Sag., 1x1x 1mm³ | Sag., 1x1x 1mm³ | Sag.,  1.33 x  1.33 x 1.33 mm³ | Sag., 0.47 x 0.47 x 0.9 mm³* | Sag., 0.9 x 0.9 x 0.9 | Sag.,  1.25 x 1.25 x 1.25 mm³ | Sag., 1x1x 1mm³ | Sag., 1x1x 1mm³ | Sag.,  1.33 x  1.33 x 1.33 mm³ |

*) interpolated, acquisition was with 0.9375 x 0.9375 x 0.9 mm³. vfl = variable flip angle.
